# Altered behavior and digestive outcomes in adult male rats primed with minimal colon pain as neonates

**DOI:** 10.1186/1744-9081-4-28

**Published:** 2008-07-10

**Authors:** Jing Wang, Chunping Gu, Elie D Al-Chaer

**Affiliations:** 1 Center for Pain Research, Departments of Pediatrics, Internal Medicine, Neurobiology and Developmental Sciences, University of Arkansas for Medical Sciences, Little Rock, AR, USA

## Abstract

**Background:**

Neonatal colon irritation (CI; pain or inflammation) given for 2 weeks prior to postnatal day 22 (PND22), causes long-lasting functional disorders in rats that can be seen 6 months after the initial insult. This study looked at the effect of varying the frequency and duration of neonatal CI on the rate of growth, digestive outcomes, exploratory activity, and colon and skin sensitivity in adult rats.

**Methods:**

Male Sprague-Dawley rats were given CI using repeated colorectal distension (CRD) at different time intervals and for varying durations starting at PND 8, 10 or 14. Control rats were handled by the investigator without any intracolonic insertion. Further experiments were done on adult rats. Digestive outcomes (food and water consumption, fecal and urinary outputs) were measured using metabolic cages. Exploratory behavior was measured using digital video tracking in an open field. Cutaneous sensitivity was assessed by measuring the responses to mechanical and heat stimuli applied to the shaved abdomen or hind paws. Visceral sensitivity was measured by recording electromyographic responses, under light isoflurane anesthesia, from the external oblique muscles in response to CRD.

**Results:**

No significant weight differences were observed between CI and control rats. Exploratory behavior was reduced in rats with neonatal CI compared to control. Digestive outputs and somatic and visceral sensitivity changed between different treatment groups with earlier and more frequent insults yielding a higher deviation from normal.

**Conclusion:**

The diversity of behavioral and digestive symptoms in these rats parallels the diversity of symptoms in patients with functional gastrointestinal disorders and is consistent with global plastic changes affecting more than one system in the organism.

## Background

Level of pain sensitivity, efficacy of analgesics, and susceptibility to developing chronic pain conditions are all subject to individual variability. The sources of such variability can be organismic, environmental or related to personal life history. Organismic variables, such as gender, age, hormonal status, genetic variability, and interactions among these factors have been carefully studied [1-3]. Environmental factors that are extrinsic to the individual such as the social environment, stressful conditions, or light cycle have also been studied and shown to contribute to variability in pain-related traits [4,5]. However, the individual's life history with noxious stimuli has often been overlooked as a potential source of variability in an adult pain experience; yet it is one that can affect sensorimotor processing, pain sensitivity and other behavioral outcomes.

Pain experience early in life can, theoretically, shape the developing nervous system at a time when heightened plasticity characterizes early postnatal development. Chronic abdominal pain is frequently seen during infancy and is often associated with functional constipation. Functional constipation occurs in otherwise well infants at the time of weaning from breast milk to infant formula. Stools become firm with the transition to formula. If a child experiences pain in the anal sphincter while passing a large hard bowel movement, the child becomes conditioned to avoid defecation [6]. The propagating colonic contractions push against an obstructed anal sphincter with pressures of 80 mm Hg and more, well above the threshold for colorectal pain [7]. Colonic painful distension in neonatal rats mimics the naturally occurring pressure build-up in the descending colon and rectum of human infants and provides a reproducible and controllable nociceptive visceral procedure. Most studies have looked at the adult sequelae of child stress and abuse and have focused on emotional and psychological problems, defects in interpersonal relationships, sexual maladjustment and social function [8-10]. Few studies have suggested an association between a history of physical abuse and functional gastrointestinal disorders [11], particularly irritable bowel syndrome (IBS) [12-14]. On the other hand, a number of studies have suggested that sometimes well-intended but painful medical procedures on neonates, without or with inadequate anesthesia, can have negative long-term implications and can engender unwanted consequences [15,16]. In a cohort matched case-control study, using siblings as controls, noxious stimulation caused by gastric suction at birth was associated with an increased prevalence of functional intestinal disorders in later life, possibly linked to the development of long-term visceral hypersensitivity and cognitive hypervigilance [17].

Experimentally, repetitive exposure of neonatal rats, over a period of two weeks, to colon pain using colorectal distension or colon inflammation with mustard oil caused long-term visceral hypersensitivity measurable six months after the initial injury [18]. This hypersensitivity was associated with central and peripheral neural sensitization [19]. Gastric suctioning during the neonatal period also resulted in global chronic somatic and visceral hyperalgesia in adult rats [20]. Similarly, exposure to painful foot-shock in the pre-weanling period had a long-term effect on the sensitivity of rats to painful events [21]. In adult rats exposed to a brief period of inflammation just after birth, the skin receptive field supplied by individual dorsal horn neurons decreased by more than 30% [22], implying permanent alterations in the spinal pain processing for these areas. Short-lasting local inflammation (produced by injection of 0.25% carageenan), produced a long-term hypoalgesia at baseline, which occurred equally in the previously injured and uninjured paws [23]. However, after re-inflammation, a long-term hyperalgesia occurred in the neonatally-injured paw, indicating a significant segmental involvement in the spinal processing of pain [24]. Despite some discrepancies in the results of these studies, most of them indicate that brief or repetitive pain exposures during early periods of development can have a long-term effect on the behavior of the adult.

This study focuses on the effect of variability in the individual's life history on adult digestive function and experience of pain. It examines the effect of neonatal painful colon distension, applied with varying onset time, duration and frequency, on the rate of growth, digestive outcomes (food and water consumption, fecal and urinary output), spontaneous exploratory behavior and visceral and somatic sensitivity of adult rats. Preliminary results were previously reported in abstract form [25].

## Methods

### Animals

Experiments were done using male Sprague-Dawley rats obtained as pre-weanling neonates (younger than 6 days) from Harlan Sprague-Dawley Inc. (Indianapolis, Indiana). They were housed in plastic cages containing corn chip bedding (Sani-Chips, PJ Murphy Forest Products, Montville, NJ) and maintained on a 12:12 h light:dark cycle (lights on at 07:00 h). The irritation procedure and the experimental testing were conducted during the light component of the cycle. The neonates were housed 10 in a cage with 1 adult female until they were 25 days old. The adult female had access to food and water ad libitum. After separation, the male rats were housed 4 in a cage with access to food and water ad libitum. At the weight of 250 g, only two rats from the same testing group (i.e. control or mechanically irritated) were together in any cage. All studies were performed in accordance with the proposals of the Committee for Research and Ethical Issues of the International Association for the Study of Pain [26] and were approved by the Institutional Animal Care and Use Committee at the University of Arkansas for Medical Sciences in accordance with the guidelines provided by the National Institutes of Health, USA.

### Neonatal colon irritation

Neonatal rats (8 – 21 days) were exposed to mechanical irritation of the colon of variable duration and at different ages according to the following protocol:

#### Neonatal colon irritation

Male Sprague-Dawley rats (8 days old) were divided into 2 groups for purposes of different treatments. Group 1 received colorectal distension (CRD) between the ages of 8 and 21 days at different time intervals and for varying durations and frequencies. The distension was applied using angioplasty balloons (Advanced Polymers Inc., length: 20.0 mm; diameter: 3.0 mm) inserted rectally into the descending colon. The balloon was distended with 0.3 ml of water, exerting a pressure of 60 mmHg (as measured with a sphygmomanometer), for 1 minute and then deflated and withdrawn. The distension was repeated 2 times (separated by 30 minutes) within an hour. This group is referred to as the group with neonatal colon irritation (CI).

Group 2 was handled in a way similar to group 1 except that no colonic insertion was made. In this group, rats between the ages of 8 and 21 days were separated from their mothers for periods of time equal to the corresponding maternal separation in CI rats, but they were only gently held and touched on the perineal area. Group 2 served as control.

The neonatal time period during which the 2 groups were irritated or handled, as well as the duration and frequency of the irritation protocol was varied consistently among the two groups in order to establish a timeline for the onset of long-lasting colon hyperalgesia in these rats. For onset variation, CI was started either on PND8, PND10, or PND14 (PND21 was tested earlier [18]). Duration indicates how long (number of days) the irritation was repeated: 1, 3, or 7 days (we reported earlier the effect of a daily 14 day irritation protocol [18]). Frequency indicates how often the irritation was given, and it varied from daily to every other day. During this period, rats from each group were housed in cages with their mothers. No treatment, procedure or further intervention was done by the investigator for 4 weeks after PND21.

### Food and water intake and changes in fecal and urine outputs

Fecal and urine output collections as well as water and food consumptions were evaluated using metabolic cages with wire mesh bottoms (Nalge Company, NY). Adult rats were individually housed (1 rat/cage) for 5 days (2 days for acclimation to the metabolic cage and 3 days for fecal collection). The final fecal output data is the average of fecal collection in the last 3 days. Fecal pellets were collected into a plastic bottle located at the bottom of the cage. The cage is designed to prevent water content in fecal pellets from evaporation and also to prevent urine from getting mixed with feces. The fecal pellets were collected daily, immediately weighed, then baked on a hot plate for two and half hours (95°C) until completely dried. The dried fecal pellets were weighed again. The water percentage contained in the fecal pellets was calculated as follows: [(Weight of fecal pellet before baking) – (weight after baking)]/(Weight of fecal pellet before baking).

The method was further standardized using the following two steps: 1) adjusting the output weight to the rat body weight by dividing the output weight by the rat body weight and obtaining thereby an output weight per gram body weight; and 2) multiplying the (output weight)/(gram body weight) for each rat by a factor of 280 which represents the median weight of all the rats used. Food and water intake were quantified by measuring the amount of food (weight of pellets in g) and water (volume in ml) consumed over a defined period of time and adjusting it to body weight. Besides fecal output, daily urine discharge (ml), daily water (ml) and food (g) consumption, and daily rat body weight were also measured to monitor the daily digestive status of the rats. The data collected from individual CI animals were compared to control; those that fell within control range were described as normal; those that fell outside the control range were described as decreased or increased.

### Behavioral testing

#### Testing spontaneous exploratory behavior

To measure spontaneous exploratory behavior, we used 4 separate activity enclosures or arenas (San Diego Instruments, CA). The arenas (each 50 × 50 cm^2^) were made of opaque plastic. A PC-linked digital video camera was mounted above the arenas. Each arena was virtually divided into 16 zones using SMART software (Panlab, Spain). Four main parameters were measured: 1) total distance traveled across the arena (cm), 2) average maximum speed at which the animal traveled, 3) number of entries the animal made into a different zone, and 4) total resting time. The enclosures were thoroughly cleaned both before and after each testing period. Animals were always tested at the same time during the day (10 a.m.) in a separate room where no other people or animals were present, with a low noise level and controlled temperature (70–72°F). The activity was recorded over a period of 2 hours for each rat. However, rats tended to become idle after 45 minutes in the arena; therefore, the data was analyzed at 5 minute time point intervals for the first 45 minutes and then compared between CI and control rats.

#### Testing somatic sensitivity

All somatic sensitivity experiments were conducted by an investigator blinded to the type of rat (control or CI). Somatic sensitivity was assessed by measuring the paw or abdomen withdrawal latency to radiant heat as a measure of secondary heat hyperalgesia according to the protocol of Hargreaves et al. [27], or the response to von Frey hair stimulation as a measure of mechanical hyperalgesia. To quantitatively assess the nociceptive threshold to radiant heat of the hind paw, animals were placed in clear plastic cages on an elevated glass plate. The rats were allowed to acclimate for 30 min before testing. A mobile radiant heat source located under the glass was focused onto the hind paw of the rats. The source focused a high intensity light beam through the glass plate onto the plantar surface of the hind paw until the rat lifted its paw. The paw-withdrawal latency (PWL) was recorded by a digital timer. Both hind paws were tested independently (five trials per side; 5-min intervals between trials). The withdrawal latencies for the left and right paws were averaged independently, and the mean value was used to indicate the sensitivity to noxious heat stimulation. The apparatus was adjusted at the beginning of each individual rat study so that the baseline PWL was approximately 10 seconds (s). This setting (i.e., the light beam intensity) was kept unchanged for the remainder of the study. The cut-off of 30 s was used to prevent potential tissue damage.

For abdominal withdrawal latency (AWL), the abdomens of tested rats were shaved and a protocol similar to the one described for PWL was adopted. The light beam was shone on a point on a previously marked area of the lower abdomen, right above the virtual intersection of two imaginary lines extending from the sternum caudally and the lowest rib ventrally. Similar to visceral sensitivity, individual data from CI rats were compared to the mean data from the control group plus or minus two standard deviations (SDs). Individual rats were considered hypersensitive to heat, if PWL or AWL was shorter than the control mean PWL or AWL, respectively, minus two SDs.

The threshold for mechanical hyperalgesia was measured by using a series of calibrated von Frey hairs (Semmes-Weinstein, Stoelting, IL). The plantar surface of the hind paw was touched with different von Frey hairs with a bending force of 0.217–12.5 g. Ten trials were done on each paw. If the rat responded to the stimulation by withdrawing the paw 5 times out of the 10 trials then it was taken as a threshold. If the rat responded to the stimulation by withdrawing the paw more than five times, the next weaker hair was used until the threshold was found. To avoid excessive stimulation, the testing was started in the following sessions with the weakest hair that had elicited withdrawal responses in the previous session. Mechanical hyperalgesia was determined by comparing the number of withdrawal responses out of 10 trials in CI rats versus the number of withdrawal responses in the control rats. A similar protocol was adopted in testing mechanical hyperalgesia on the abdomen.

#### Testing visceral sensitivity

Behavioral responses to colorectal distension (CRD) were assessed in all groups around the age of 3 months by measuring the visceromotor reflex (VMR). The VMR is a reflex measured using an electromyographic (EMG) recording obtained from the external oblique muscle. VMR was recorded in adult rats sedated by light isoflurane anesthesia (2%). While sedated, the rats exhibited no voluntary movements but showed reflexive responses to nociceptive stimuli. The EMG electrode (Teflon-coated stainless steel wire) was inserted through a small skin incision into the external oblique muscle superior to the inguinal ligament and was connected to an amplifier. The signal was displayed on an oscilloscope and fed into a computer using CED 1401 plus and was recorded using Spike 2 software. The raw EMG signal is biphasic and was therefore rectified using "Rectify" script in Spike 2. The rat colons were distended for 20 s every 4 min, and the rectified signal was integrated over the 20 s stimulation period to give a mean response frequency. The mean frequency of the preceding 20 s was subtracted from the distension value to give the EMG intensity as data points. Since these are multiunit recordings, the magnitude of the EMG varied between animals. The data from each animal were normalized to a baseline response derived from the mean of 3 distensions for each intensity of CRD. All subsequent data collected from the same animal were compared to their baseline values. Measuring the threshold intensity of CRD consisted of recording the stimulus intensity that evoked a VMR recorded electronically. CRD was applied in increments of 10 mmHg starting at 10 mmHg (the smallest distinguishable mark on the sphygmomanometer gauge). Comparisons of the responses of CI rats were made to those of control rats in 2 ways: 1) the average response of a group of rats with similar neonatal treatment was compared to the average response of control rats, and 2) the individual normalized data of each rat was compared to the average response of control rats to determine whether the CI rat was hypersensitive, hyposensitive or normo-sensitive. Individual rats whose responses fell within two SDs of the control mean were considered normal. Rats whose responses were smaller than the control mean minus two SDs [< (Mean – 2SD)] were considered hyposensitive, and those whose responses were greater than the control mean + two SDs [> (Mean + 2SD)] were considered hypersensitive.

Rats tested with somatic stimuli were also tested with visceral stimuli and vice versa. The two types of stimuli were separated by 4 days to avoid interactive effects between stimuli.

#### Colon stimulation in adult rats

Colon stimulation consisted of graded CRD produced by inflating a balloon inside the descending colon and rectum. The balloon was 4 cm in length and made of the finger of a latex glove. It was attached to polyethylene tubing and inserted through the anus into the rectum and descending colon. The open end of balloon was secured to the tubing with thread and wrapped with tape (1 cm wide). The balloon was inserted so the thread was approximately 1 cm proximal to the anal sphincter. The balloon was held in place by taping the tubing to the tail. The tubing was attached via a T connector to a sphygmomanometer pump and a pressure gauge. Prior to use, the balloon was blown up and left overnight so that the latex stretched and the balloon became compliant. CRD was produced by rapidly inflating the balloon to the desired pressure (20, 40, 60 or 80 mmHg) for a duration of 10 s. Stimuli were consecutive (spaced by 20 s) and applied in an ascending graded manner (e.g. 3 × 20, 3 × 40 etc.).

### Statistical analysis

The data were analyzed for statistical significance using Sigma stat software. A Friedman's test was used to assess if responses changed across pressures within each group. The differences in the median values of the EMG response among the 2 treatment groups (CI and Control) at each pressure of CRD were compared using the Kruskal-Wallis (K-W) One Way Analysis of Variance on Ranks. If the K-W test was significant (p < 0.05) we did pairwise comparisons using a Wilcoxon Rank Sum test with a Bonferroni correction at 0.05/2 to correct for multiple comparisons. Stated significant results refer to a p < 0.05/2.

A One Way Analysis of Variance was made to compare the differences in the median values of the thresholds to elicit a distinctive abdominal contraction measured in the 2 groups. This was followed by pairwise comparisons using a Bonferroni t-test with a corrected p-value of 0.05/2.

For exploratory behavior and somatic sensitivity testing, the responses were compared using a One Way Analysis of Variance between CI and normal rats. Significance was determined whenever p < 0.05.

## Results

Minimal neonatal CRD in rats leads to a state of mechanical visceral and referred somatic heat hypersensitivity in adults, manifested respectively by increased contractility of abdominal muscles in response to CRD and shorter paw and abdomen withdrawal latencies in response to radiant heat. This state of hypersensitivity exists in the absence of colon inflammation. Tissue examination of sections from the colons of 24 rats (CI, n = 18; Control, n = 6) in various subgroups showed no significant structural damage or loss of crypts. Mucin depletion or increase in intraepithelial lymphocytes was not seen in any of the tissues examined. Slides from the various subgroups were rated as 1+. No significant difference in weight was seen among the adult CI subgroups and between adult CI rats and control rats of similar age. On the other hand, alterations in fecal output could be seen in a number of rats.

Varying the duration and onset time of the neonatal stimulus affected the outcome in adults: a stimulus with a later onset (after PND14) and slower frequency was less likely to evoke all the symptoms seen with an early-onset stimulus (before PND14). However, combining an early-onset with high-frequency stimulation increased the risk for undesirable side effects in the developing rat; these included loss of the animal (death) before it reached adult age, apparent damage to the anal sphincter and occasional enlargement and swelling of the colon seen during histological examination. On the other hand, reducing the frequency of the neonatal CI to one time only, even though given on PND8, did not always produce consistent hypersensitivity in adult rats and the margin of error was larger. In general, rats that received neonatal CI only once showed functional outputs (digestive and behavioral) within the normal range. Optimal combinations of onset-time and duration consisted of a stimulus given twice a day on PND8, PND10 and PND12 or PND10, PND12 and PND14 (see Tables 1, 2, 3).

**Table 1:** Summary of digestive, behavioral and histological  data obtained from adult rats with neonatal CI (onset of CI at PND8).

**TABLE 1**	PND8 (n = 8)	PND8, 9, 10 (n = 12)	**PND8, 10, 12 **(n = 44)	PND8 – PN14 (n = 12)
Survival	8	10	44 (100%)	8
Food Consumption	1 I; 7 N	4 I; 1 D; 5 N	21 I; 23 N	2 I; 1 D; 5 N
Water Consumption	8 N	3 I; 7 N	8 I; 36 N	3 I; 1 D; 4 N
Dry Feces	2 I; 8 N	5 I; 5 N	21 I; 23 N	4 I; 1 D; 3 N
Water in Feces	8 N	2 I; 3 D; 5 N	6 I; 11 D; 27 N	2 I; 2 M; 4 N
Urine Output	8 N	1 I; 9 N	7 I; 37 N	3 I; 5 N
Somatic Sensitivity	8 N	6 I; 2 D; 2N	22 I; 11 D; 11 N	6 I; 1 D; 1 N
Visceral Sensitivity	2 I; 1 D; 5 N	7 I; 2 D; 1 N	24 I; 12 D; 8 N	5 I; 2 D; 1 N
Colon Histology	N	9 N; 1*	N	5 N ; 3*

Summary of digestive, behavioral and histological data obtained from adult rats with neonatal CI (onset of CI at PND8). Somatic sensitivity refers to the abdominal sensitivity to heat. Visceral sensitivity refers to the responses to CRD (60 mmHg). Colon histology refers to the inflammation grade by comparison to control. N: normal by comparison to the range of control values. I: increased, above the range of normal values. D: decreased, below the range of control values. * indicates animal had colon abnormality (e.g. enlarged colon, relaxed anal sphincter, or other).

**Table 2:** Summary of digestive, behavioral and histological data obtained from adult rats with neonatal CI (onset of CI at PND10).

**TABLE 2**	PND10 (n = 8)	PND10, 11, 12 (n = 12)	PND10, 12, 14 (n = 12)	PND10 – PN16 (n = 12)
Survival	8	11	11	9
Food Consumption	8 N	4 I; 2 D; 5 N	5 I; 1 D; 5 N	3 I; 1 D; 5 N
Water Consumption	8 N	3 I; 8 N	2 I; 9 N	5 I; 1 D; 3 N
Dry Feces	1 I; 7 N	5 I; 6 N	4 I; 7 N	4 I; 2 D; 3 N
Water in Feces	8 N	2 I; 3 D; 6 N	3 I; 3 D; 5 N	2 I; 3 D; 4 N
Urine Output	8 N	1 I, 10 N	1 I; 10 N	3 I; 6 N
Somatic Sensitivity	8 N	7 I; 4 N	8 I; 1 D; 2 N	6 I; 2 D; 1 N
Visceral Sensitivity	2 I; 6 N	8 I; 2 D; 1 N	7 I; 2 D; 1 N	7 I; 1 D; 1 N
Colon Histology	N	N	N	5 N; 3*; 1 (lost)

**Table 3:** Summary of digestive, behavioral and histological data obtained from adult rats with neonatal CI (onset of CI at PND14).

**TABLE 3**	PND14 (n = 8)	PND14, 15, 16 (n = 12)	PND14, 16, 18 (n = 12)	PND14 – PN20 (n = 12)
Survival	8	12	12	10
Food Consumption	8 N	5 I; 7 N	4 I; 8 N	3I; 2 D; 5 N
Water Consumption	8 N	3 I; 9 N	1 I; 11 N	4 I; 2 D; 4 N
Dry Feces	8 N	3 I; 2 D; 7 N	4 I; 8 N	5 I; 2 D; 3 N
Water in Feces	8 N	3 I; 3 D; 6 N	2 I; 3 M; 7 N	2 I; 3 D; 5 N
Urine Output	8 N	2 I; 10 N	12 N	3 I; 7 N
Somatic Sensitivity	8 N	6 I; 3 D; 3 N	8 I; 4 N	6 I; 4 N
Visceral Sensitivity	1 I; 7 N	5 I; 3 D; 4 N	7 I; 1 D; 4 N	7 I; 3 N
Colon Histology	N	N	N	7 N ; 3*

### Changes in digestive outcomes

**Figure 1:**
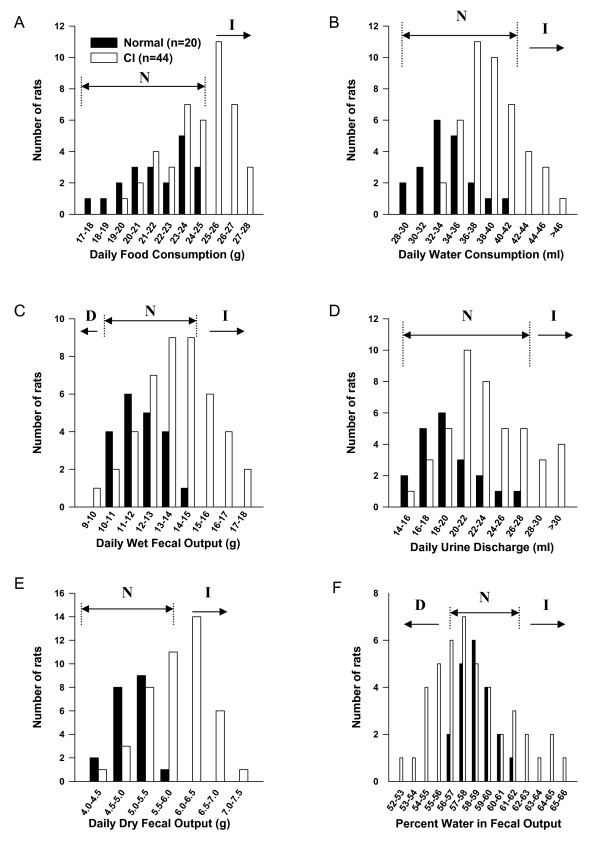
**Digestive outcomes**. Bar graphs represent the number of rats with a given digestive outcome. The graphs compare 6 measurable outputs of rats treated with neonatal colon irritation (CI; PND8, 10, 12) to control rats (Normal): **A**. Daily food consumption in g. **B**. Daily water consumption in ml. **C**. Daily wet fecal output. **D**. Daily urine discharge. **E**. Daily dry fecal output. **F**. Percent water in fecal output. N: indicates the normal range for each output. D: indicates decreased output (below normal) and I indicates increased output (above normal).

Food and water consumption and fecal and urine outputs were monitored in a total of 149 adult rats: 131 adult rats that received neonatal CRD (see Tables 1, 2, 3) and 18 control rats (12 treated on PND 8, 10 and 12; 6 treated on PND 10, 12 and 14). The results from individual rats with neonatal CI were compared to control rats. For example, the normal range for wet fecal output observed in control rats was between 10 g and 15 g (Fig 1C). Among 44 rats treated on PND8, 10 and 12, twenty-nine rats (65.9%) had normal fecal output (output fell within the normal range), 1 rat (2.3%) had decreased fecal output (between 9 g and 10 g) and 12 rats (12.3%) had increased fecal output (between 15 g and 18 g). The daily percent water in fecal output was within control range (normal) in 27 rats (61.4%), decreased in 11 (25%) and increased in 6 (13.6%) (Fig. 1F). Similar observations were made of other parameters within the same treatment group (Fig. 1) and of other treatment groups (see Tables 1, 2, 3).

### Behavioral studies

#### Spontaneous exploratory activity

**Figure 2:**
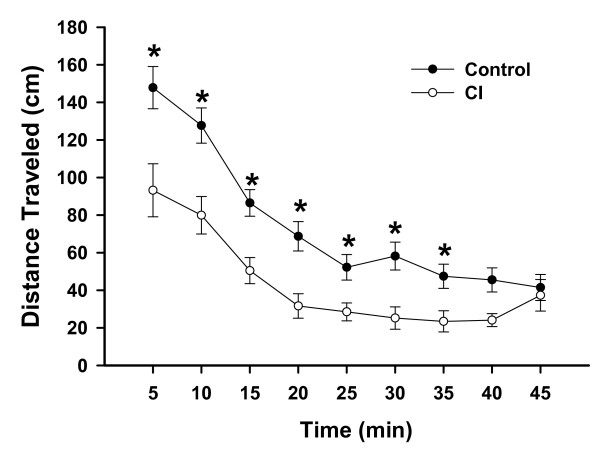
**Exploratory activity**. Line graphs represent the average distance (+/- SEM) traveled (cm) by control rats (n = 12; solid circle) and rats with neonatal CI (n = 12; open circle), calculated every 5 minutes over a period of 45 min. * means p < 0.05/2.

Spontaneous exploratory activity was tested in 24 adult rats (control, n = 12; CI, n = 12) that received neonatal treatment on PND8, 10 and 12. Among the 4 parameters measured, the total distance traveled (Fig. 2) was significantly reduced in CI rats compared to control rats. No significant differences were observed in the number of entries from one zone into another (although slightly reduced for CI during the first 25 minutes), their resting time (although it was longer for CI rats than for control rats during the first 10 minutes), or in the maximal velocity of movement between the two groups. Figure 2 illustrates the timeline and the time points at which the distance traveled was measured and shows that CI rats consistently traveled a shorter distance at any time point.

#### Somatic hypersensitivity

**Figure 3:**
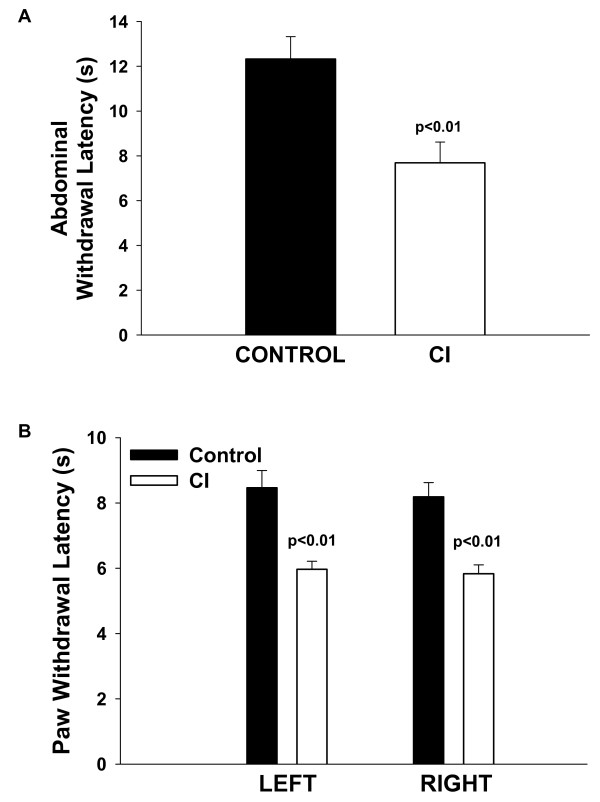
**Somatic sensitivity**. Bar graphs show the average (+/- SEM) withdrawal latency (s) to radiant heat shone on a shaved area of the abdomen (**A**) or on the hind paws (**B**) in control rats (n = 12) and rats with neonatal CI (PND8, 10 and 12; n = 22).

Somatic sensitivity was assessed in all groups by measuring the responses to mechanical and heat stimuli applied to the abdomen or the hind paws. No significant differences between CI rats and control rats were observed in the average response to mechanical stimuli, although a slight decrease in the response threshold to abdominal mechanical stimuli was seen in CI rats. By contrast, the average withdrawal latencies to heat stimuli applied to either the abdomen (Fig. 3A) or the hind paws (Fig. 3B) were significantly shorter in CI rats than control rats, indicating that CI rats show signs of heat hypersensitivity referred to the abdomen and the hind paws on both sides. Figure 3 illustrates the reduced average withdrawal latencies to radiant heat in CI rats (PND8, 10 and 12) compared to control rats measured on the abdomen (A) or on the left and right hind paws (B). Similar observations were made in the other CI groups with repeated neonatal CI, although the individual results between groups varied (see Tables 1, 2, 3, somatic sensitivity). In addition, this increased sensitivity to heat stimulation, although significant, was not consistent among all rats of the same treatment group. A number of individual rats showed no change or reduced sensitivity (increased latency) to heat stimuli when compared to control – their responses fell within the range of control responses – but on average, rats that received repeated neonatal CI (3 times or more) were more sensitive than control rats.

#### Visceral hypersensitivity

**Figure 4:**
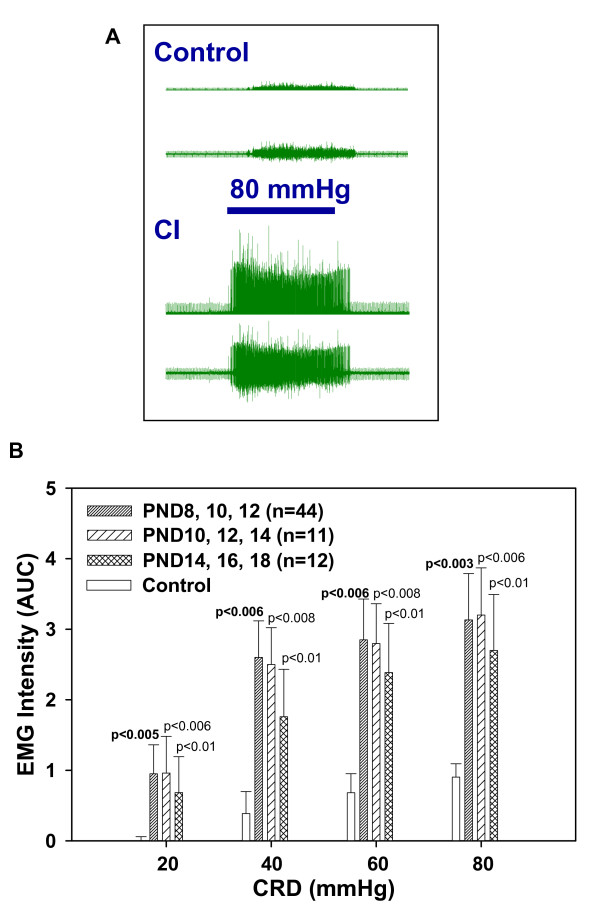
**Visceral sensitivity**. **A**. Sample waveforms recorded from the external oblique muscles in a control rat (upper panel) and a rat with neonatal CI (lower panel) in response to CRD (80 mmHg). In each panel, the lower trace represents the raw waveform and the upper trace represents the rectified one. **B**. Bar graphs represent the average (+/- SEM) EMG intensity (AUC: area under the curve) recorded in response graded CRD (20, 40, 60 and 80 mmHg) in control rats (n = 12) and in adult rats with neonatal CI given at different time points. All data are compared to control. Significance (or lack thereof) is indicated by the value of p shown for each graph. p < 0.05/2 is significant.

One hundred and thirty (130) adult rats (age 3 months) treated with neonatal CI were tested for colon hypersensitivity by measuring their responses to CRD using EMG measurement of the abdominal muscle contractions. A majority of adult rats treated with neonatal CI showed visceral hypersensitivity (increased responses to CRD compared to control), with some showing reduced sensitivity and others normal sensitivity (see Tables 1, 2, 3, Visceral Sensitivity). Except for adult rats treated with a single neonatal CI, the average EMG responses to CRD were higher in CI rats than in control (Fig. 4). This visceral hypersensitivity was most significant in rats that received repeated neonatal CI starting at PND8 or PND10. Repetition of neonatal CI for at least 3 times, (e.g. on PND8, PND10 and PND12) yielded a more dramatic increase in visceral hypersensitivity than a single episode (e.g. PND8). However repetition of neonatal CI for more than 3 days (e.g. PND8-PND14) often caused a number of undesirable effects including a higher incidence of pup death. Less significant hypersensitivity was noted with later onset and fewer number of neonatal CI (e.g. onset on PND14 or 1 time CI on PND8).

When individual responses to CRD of adult rats treated with neonatal CI were compared to the range of individual responses to CRD among control rats, variations in individual visceral sensitivity (increased, decreased or normal) were seen in all groups (see Tables 1, 2, 3, visceral sensitivity). Optimal combinations of onset date and duration were found to be: a) stimulus started on PND8 and repeated on PND10 and PND12; or, b) stimulus started on PND10 and repeated on PND12 and PND14. These combinations of onset and duration minimize the risk of loss of the developing rats while maximizing the significance of relevant symptoms they express as adults compared to controls. For example, adult rats that received neonatal CI at PND8, PND10 and PND12, had a 100% survival rate, increased visceral and somatic sensitivity in addition to a full spectrum of fecal output ranging from reduced to normal to increased (see Tables 1, 2, 3).

**Figure 5:**
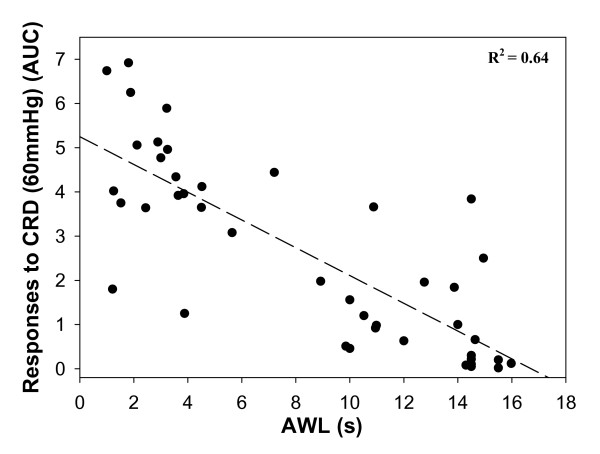
**Correlation of visceral and somatic sensitivity**. Linear regression curve illustrates the correlation between somatic (AWL) and visceral (CRD = 60 mmHg) sensitivity in CI rats. R^2 ^= 0.64.

Visceral sensitivity correlated with somatic sensitivity but not with digestive outcomes in rats treated with neonatal CI. For example in rats that received neonatal CI on PND8, 10 and 12, responses to CRD (60 mmHg) correlated with the AWL with a correlation coefficient of 0.64 (Fig. 5). Similar correlations were observed between the AWL and responses to CRD (40 and 80 mmHg) for this and other groups. But, the correlation was weak between the PWL and the responses to CRD with maximum r^2 ^= 0.34 observed between CRD (60 mmHg) and the PWL in the PND8, 10, 12 group.

## Discussion

The main observation made in this study is that limited neonatal irritation to the colon in rats can lead to significantly increased responses to CRD in adults and significantly shorten withdrawal latencies in response to heat stimulation of the abdomen or the hind paws. These observations are consistent with chronic visceral hypersensitivity and increased somatic sensitivity and mimic to a large extent symptoms of chronic abdominal pain with pain referred to somatic structures in humans. In addition, the neonatal insult can reduce exploratory behavior in adult rats and alter their fecal outcome, despite the absence of colon inflammation, mimicking thereby symptoms of discomfort and altered stools in some patients with IBS. These sensory and digestive functional outcomes are, on average, significantly higher in rats with neonatal CI than in control rats. A closer look at the results from individual rats with neonatal CI reveals that, compared to control, the outcomes vary from one animal to the next despite the uniformity of the neonatal insult. This variability is seen in behavioral responses to visceral and somatic stimuli, and also in digestive outputs. For example, when neonatal rats were exposed to the same amount of colon pain, some of them became hypersensitive adults, others became hyposensitive and some did not show any changes in sensory sensitivity. Similarly, fecal output and other digestive parameters increased in some adult rats exposed to neonatal pain, but decreased or stayed within normal ranges in some others. The study offers evidence of individual variability in the pain response and for the first time the possibility of modeling a syndrome of variable symptoms in rats.

Another cardinal observation of this study is that the effects of the neonatal injury were global. They involved somatic and visceral sensory processes (which affect pain-related behaviors), arousal/affective processes (which affect spontaneous exploratory behavior), and digestive processes (which affect fecal and urinary outputs). These observations indicate that a number of plastic changes have taken place in more than one segment of the central nervous system and more likely in more than one system in these rats.

### Impact of varying the neonatal injury on digestive outcomes

The best way to describe the effects of repeated neonatal CI on digestive outputs would be as an expansion of the range of outputs beyond those of control animals. The digestive outputs of rats with neonatal CI were analyzed by comparing individual data from the CI rats to the range of normal data obtained from control rats. This approach yielded three different groups of CI rats – rats with normal, increased or decreased outputs – and preserved the statistically significant differences in the average digestive outputs between the groups. If the 3 groups were lumped together, the averaged data would mask individual variability and would show a small, albeit sometimes significant, (one-directional) increase in digestive outputs among all CI rats, instead of the bidirectional shift observed in reality.

This bi-directional shift in outcomes may be caused by a number of factors including: 1) structural or genetic differences between the individual rats that render some rats more or less susceptible to a particular injury or insult – such differences have been known to differentially affect behavioral outcomes [28]; 2) different social ranks among the rats of the same colony (in the same cage) that would render the dominant rats less stressed than the less dominant ones and may affect their behavior and digestive outcomes [29]; and, 3) alternating increase and decrease in outputs of the same rat that are difficult to detect when taking a momentary snapshot. These alternations may be caused by a number of factors, among them altered neural control of GI function at all levels of the nervous system (enteric, peripheral and central) or altered stress response caused by plastic changes in the hypothalamic-pituitary-adrenal axis. Pain in neonates is known to be always stressful [30], and neonatal stress can have a severe impact on adult behavior including increased fecal output, anti-social behavior, neuropsychiatric disorders and other problems in the heart and the gastrointestinal systems [31].

On the other hand, the increases in dry fecal output and in urine output correlated with increased food and water consumption and no change in weight. These correlations seem intuitively simple but are also indicative of a global effect of the neonatal injury. Despite the localization of the neonatal injury to the colon, its functional effects are trans-systemic and are seen throughout the GI tract and urinary system.

### Impact on pain-related behaviors

Varying the onset-time, duration or frequency of the neonatal insult differentially affected visceral and somatic sensitivity. Early onset and increased duration and frequency caused the rats to become more hypersensitive and their responses to colorectal distension to become more vigorous. Furthermore, their sensitivity to visceral stimuli correlated well with their sensitivity to somatic stimuli, indicating the involvement of a central mechanism in this sensitivity [18]. These observations were aggravated further the earlier the insult was begun and tended to become less pronounced with later onsets of injury; thus confirming earlier observations that injuries begun after PND21 are unlikely to cause any long-term deficits in neural, behavioral or other functional outcomes [18]. These long-term behavioral changes are related in part to global plastic changes in the sensory systems at more than one level. In fact, plastic neural changes involving adult rats with neonatal injuries have been reported to affect primary afferents innervating the periphery [32], spinal neurons in multiple segments of the cord [18,33]), and thalamic structures and ascending and descending pathways [24,34]. These changes may also affect vagal innervation of the viscera [35], the enteric nervous system and the hypothalamic-pituitary-adrenal "stress" axis [36,37]. The latter may explain the shift in the response to stress in adult rats exposed to neonatal adversity [38-40] and possibly underlies the changes in their digestive outcomes and exploratory behavior.

A distinctive observation of these studies is the hyperalgesia seen in response to heat but not to mechanical stimulation. The lack of mechanical hyperalgesia may be related to the possibility that the mechanical stimulus (probing with von Frey filaments) activates both large and small diameter fibers, which may have been differentially sensitized by the neonatal injury. By contrast heat stimulation activates mainly small unmyelinated fibers. The development of heat hyperalgesia in the hind paws in response to neonatal visceral pain is consistent with observations made in other models of neonatal injury. For example, neonatal gastric suctioning caused visceral hyperalgesia with heat hyperalgesia in the hind paws of adult rats [20]; however, there was no indication of mechanical hyperalgesia or allodynia. In addition, a clear difference has often marked the neural mechanisms of the two types of somatic hypersensitivity. Differentiation between the responses to mechanical and heat stimuli have been widely reported in animal models of pain. For example, NMDA receptors have been shown to mediate heat hyperalgesia but not mechanical allodynia in a rat model of nerve injury; the NMDA antagonist dextrorphan reversed heat hyperalgesia but not tactile allodynia [41,42]. In a recent study using a murine ex vivo somatosensory preparation, the response characteristics of cutaneous sensory neurons staining positively for TRPV1 or TRPV2 were examined. The results suggested that TRPV1 may be essential for heat transduction in a specific subset of mechanically insensitive cutaneous nociceptors and that this subset may constitute a discrete heat input pathway for inflammation-induced thermal pain [43]. Similar differential mechanisms may underlie the sensory divergence in the neonatal colon injury model; however, these remain to be investigated.

The susceptibility of the neonatal organism to painful stimuli may relate to the novelty of the painful stimulus in a neonatal context. The nociceptive neuronal circuits are generally formed during embryonic and postnatal times when painful stimuli are normally absent or limited, but the descending inhibitory pathways, which normally control the flow of nociceptive signals through the spinal cord to higher brain structures, do not mature until later in development [44], leaving the door open for uncontrolled pain signals to wreak havoc in higher brain structures. Therefore, the occurrence of pain during this period is likely to have a more dramatic effect on the plasticity of the nervous system [45,46] and to impact development across several critical time points. It is also likely to be responsible for some of the devastating and permanent behavioral outcomes and disruptions in normal brain activity [47].

On the other hand, the hyposensitivity seen in a subset of neonatally injured rats is in striking contrast with the hypersensitivity seen in a different subset, but consistent with the hyposensitivity reported in other models of neonatal injury [23]. It may be related to a myriad of factors, including 1) desensitization of primary afferents caused by permanent damage during the neonatal period, 2) changes in the sensitization of the pain pathways in the CNS whereby desensitization (similar to long term depression) may have taken effect; or, 3) the triggering of a desensitization response, in what may appear to be an "inoculation" against pain, by neuroimmune factors reported to be involved in the immediate sensitization following injury. It remains to be determined why rats of the same gender, strain and breed respond differently over time to the same neonatal stimulus.

### Impact on spontaneous behavior

Spontaneous behavior was assessed by measuring exploratory activity in an open field. By comparison to control rats, rats with neonatal CI showed a decrease in the total distance traveled and the number of entries from one virtual zone into another in the open field, and their resting time was longer than that of control rats; however, there was no significant difference in the maximal velocity of movement between the two groups. These observations correlate with reduced exploratory activity. Voluntary exploratory behavior of animals in a new environment may be used as a measure of discomfort that may be associated with ongoing pain [48], distress and anxiety [49], socio-sexual behavior [50], or adaptation to or fear of leaving a familiar place, otherwise known as agoraphobia. The most common psychiatric disorders observed in IBS patients are major depression, panic disorder, social phobia, generalized anxiety, posttraumatic stress and somatization [51]. Walker et al [52] observed 412 cases of probable IBS (with no other pain problems) and reported that individuals with IBS showed higher rates of major depression (13.4%), panic disorders (5.2%) and agoraphobia (17.8%). Whereas agoraphobia in IBS patients is believed to be caused in most cases by the fear of "not finding a bathroom on time" and can impair a person's lifestyle and career, an organic basis for agoraphobia or an association of agoraphobia with childhood abuse cannot be excluded [53,54]. Studies are underway to determine whether these rats are suffering from depressive or anxiety-like disorders.

## Conclusion

Adverse experiences in the neonatal period often contribute to adaptive or maladaptive function of the mammalian nervous system, and possibly to the development of chronic intractable disorders that may occur in the absence of identifiable structural problems and which can sometimes be very painful, hence the cognomen 'functional pains' (e.g. fibromyalgia, functional abdominal pain, etc.). These pains can be associated with other functional disorders, can become the consuming focus of a patient's life, and may be onerous to the treating clinician, particularly in the absence of a traceable etiology. This study has shown that an individual's life history with noxious stimuli is a potential source of variability in adult pain experiences and one that can affect sensorimotor processing, pain sensitivity and other behavioral outcomes. In addition, it has shown that a cluster of symptoms as diverse as those observed in irritable bowel syndrome may be modeled in animals. Limited neonatal injury in rats can yield adult outcomes that mimic symptoms of functional GI disorders in man. These symptoms occur in the absence of colon inflammation and include visceral hypersensitivity with referred somatic pain, altered fecal output and reduced exploratory behavior. Whereas the sensitivity can be explained by sensitization of the nervous system, the underlying causes of altered fecal output and reduced exploratory behavior remain to be determined and point to a global phenomenon affecting the organism as a whole rather than just a single system. This is commensurate with the diversity of symptoms in functional GI disorders and the ambiguity of its pathophysiology.

## Competing interests

The authors declare that they have no competing interests.

## Authors' contributions

JW was responsible for the behavioral studies and analysis of related data. CG was responsible for treatment of neonatal rats, digestive outcome studies and analysis of related data. EDA-C was responsible for experimental design, data analysis, statistics, and overall project administration. All authors contributed to the writing of the manuscript.
